# Use of Complementary and Alternative Medicine in the Management of Breast Cancer

**DOI:** 10.1001/jamanetworkopen.2026.0337

**Published:** 2026-03-02

**Authors:** Oluwaseun F. Ayoade, Giorgio Caturegli, Maureen E. Canavan, Benjamin J. Resio, Elizabeth R. Berger, Daniel J. Boffa

**Affiliations:** 1 Division of Thoracic Surgery, Department of Surgery, Yale University School of Medicine, New Haven, Connecticut; 2 Division of Surgical Oncology, Department of Surgery, Yale University School of Medicine, New Haven, Connecticut; 3 Yale Cancer Outcomes, Public Policy and Effectiveness Research Center, Yale University School of Medicine, New Haven, Connecticut

## Abstract

**Importance:**

Innovations in both the surgical and medical management of breast cancer over the past few decades have led to reductions in treatment-related morbidity and increases in overall survival. Despite these advancements in traditional therapies, including surgery, chemotherapy, radiation, endocrine therapy, and immunotherapy, a subset of patients continues to choose complementary and alternative medicine (CAM).

**Objective:**

To describe the association of CAM with survival in patients with breast cancer.

**Design, Setting, and Participants:**

This cohort study analyzed data from the National Cancer Database on female patients diagnosed with breast cancer from 2011 through 2021. Survival time was compared among patients who received traditional treatment, traditional treatment and CAM, and no treatment. Data were analyzed from May 2025 to December 2025.

**Exposure:**

Complementary or alternative treatment, defined in the NCDB as treatment administered by nonmedical personnel.

**Main Outcomes and Measures:**

The primary outcome was 5-year survival. Unadjusted 5-year survival was assessed by Kaplan-Meier analysis, and adjusted survival was assessed with a Cox proportional hazards model controlled for age, race and ethnicity, Charlson Comorbidity index, insurance type, facility type, region, year of diagnosis, cancer stage, and income.

**Results:**

Of 2 169 202 female patients with breast cancer identified, 2 157 219 (median [IQR] age, 62 [52-71] years) were included in the sample. A total of 2 106 665 patients (97.6%) received traditional therapy, 273 (<0.1%) received CAM alone, 568 (<0.1%) received a combination of CAM and traditional therapies, and 49 713 (2.3%) received no treatment. Compared with patients treated with traditional therapies, those treated with CAM alone (adjusted hazard ratio [AHR], 3.67; 95% CI, 3.03-4.44; *P* < .001) or no treatment (AHR, 3.53; 95% CI, 3.48-3.58; *P* < .001) had the highest risks for mortality. Patients who received a combination of traditional therapies and CAM were less likely to receive endocrine therapy (eg, 40.7% vs 65.2% in stage II; *P* < .001) and radiation (59.5% vs 36.6% in stage II; *P* < .001) compared with patients treated exclusively with traditional therapies. Receipt of a combination of traditional therapies and CAM was associated with a higher mortality compared with being treated exclusively with traditional therapy (AHR, 1.45; 95% CI, 1.22-1.72; *P* < .001).

**Conclusions and Relevance:**

In this cohort study of data from female patients with breast cancer included in the NCDB, the use of CAM instead of traditional therapies was uncommon but was associated with a reduction in survival time. Further study is warranted.

## Key Points

**Question:**

What is the association of complementary and alternative medicine (CAM) with survival in female patients with breast cancer?

**Findings:**

In this cohort study involving 2 157 219 women with breast cancer, those who combined traditional therapies, such as surgery, chemotherapy, radiation, endocrine therapy, and immunotherapy, with CAM therapies were less likely to receive endocrine therapy and radiation compared with patients treated exclusively with traditional therapies. Combination of CAM and traditional therapies was associated with higher mortality compared with being treated exclusively with traditional therapy.

**Meaning:**

Findings of this study suggest that the use of CAM instead of traditional therapies could be associated with a reduction in survival in breast cancer, and further study is warranted.

## Teaser

This cohort study assesses the association of complementary and alternative medicine plus traditional therapies, including surgery, chemotherapy, radiation, endocrine therapy, and immunotherapy, with survival in female patients with breast cancer.

## Introduction

Breast cancer is the second most commonly diagnosed cancer among women in the US and the second leading cause of cancer deaths in women after lung cancer.^1,2^ Several groundbreaking advancements in both early detection and treatment of breast cancer have been associated with a reduction in mortality and morbidity. Screening mammography over the past 30 years has reduced breast cancer–related mortality by 20% to 30% due to earlier cancer detection.^3^ Additionally, advancements in targeted therapy, such as selective estrogen receptor modulators in hormone receptor–positive breast cancer and use of anti-human epidermal growth factor receptor 2 monoclonal antibody in *EBBR2* (formerly *HER2*)–positive breast cancer, have contributed to the decrease in mortality^4^ The use of adjuvant endocrine therapy in hormone receptor–positive breast cancer has been associated with decreased recurrence and improved survival.^5,6,7^

Use of complementary and alternative medicine (CAM) for breast cancer is a long-standing practice, with 1 study from 1999 reporting that 28% of patients with early-stage breast cancer received CAM.^8^ More recent studies suggest that interest in CAM among patients with breast cancer continues and may be increasing.^9,10^ The most commonly used CAM modalities among patients with breast cancer are dietary supplements, mind and body approaches, and acupunture.^11^ Typical motivations for CAM use include symptom management, general disease prevention, and immune enhancement.^12^ Younger age, female sex, higher educational background, and lower socioeconomic status have all been described as factors that play a role in CAM use among patients with cancer.^13,14,15^ Certain behaviors and mindsets, including religious and or spiritual beliefs, natural and or holistic values, and distrust in the health care system, have been associated with a higher likelihood of selecting CAM over traditional therapies, including surgery, chemotherapy, radiation, endocrine therapy, and immunotherapy.^16^ Moreover, CAM use has been growing faster in racial and ethnic minority groups and in patients with barriers to accessing traditional therapies.^17,18^

Given the advancements in traditional therapies, it is particularly important to examine the outcomes associated with CAM in the modern era. The National Cancer Database (NCDB) contains information about cancer care, capturing about 70% of newly diagnosed cancers in the US and serves as an important resource for outcomes-based research.^19,20^ We evaluated trends in the use of CAM for female patients diagnosed with stage I to IV breast cancer between 2011 and 2021 in the NCDB. Our objective was to describe the association of CAM use with survival in patients with breast cancer.

## Methods

### Data Source

The NCDB is a database of oncology patients developed through a collaboration between the Commission on Cancer of the American College of Surgeons and the American Cancer Society.^19^ The NCDB Participant User File for 2022, which contains deidentified patient information, was used for the purpose of this study. Female patients who were diagnosed with breast cancer from 2011 through 2021 were included. The 11 983 patients who were missing treatment information or who were missing values for traditional therapy and/or CAM were excluded. A sensitivity analysis did not identify any obviously important differences between this group and the study population. The study was approved by the Yale University Institutional Review Board, and patient consent was waived due to use of deidentified data. The Strengthening the Reporting of Observational Studies in Epidemiology (STROBE) guideline was followed.

### Variables

Treatment was considered in the following 4 groups: traditional therapy only, CAM only, combination of traditional therapy and CAM (hereafter combination cohort), and no treatment. Traditional therapies included surgery, chemotherapy, radiation, endocrine therapy, and immunotherapy, and patients in the traditional therapy cohort received exclusively traditional therapies. The CAM cohort was treated exclusively with alternative therapies (defined in the NCDB as therapy administered by nonmedical personnel). The combination cohort received at least 1 traditional therapy and at least 1 CAM therapy. The no treatment cohort did not receive either CAM or traditional therapies.

Race and ethnicity were included as a covariate due to known differences in treatment patterns in breast cancer.^21^ In the NCDB, race and ethnicity are abstracted from the electronic health record and represents self-reported identification. As previously described, race and ethnicity were classified as follows: American Indian or Alaska Native, non-Hispanic Asian (hereafter Asian), Hispanic, Native Hawaiian or Pacific Islander, non-Hispanic Black (hereafter Black), non-Hispanic White (hereafter White), and other (non-Hispanic individuals not named in other categories).^22^ Asian race included Asian Indian or Pakistani not otherwise specified (NOS), Asian Indian, Pakistani, Micronesian NOS, Chamorro/Chamoru, Chinese, Fiji Islander, Filipino, Guamanian NOS, Hmong, Japanese, Kampuchean (Cambodian), Korean, Laotian, Melanesian NOS, New Guinean, Polynesian NOS, Samoan, Tahitian, Thai, Tongan, Vietnamese, and Other Asian including Asian NOS and Oriental NOS. Hispanic included Cuban, Dominican, Mexican (including Chicano), Puerto Rican, South or Central American (except Brazilian), other specified Spanish/Hispanic origin includes European and excludes Dominican, Hispanic NOS, Latino NOS, Spanish NOS, and Spanish surname only. Additional covariates included age (18 to 49, 50 to 64, 65 to 74, and ≥75 years), Charlson Comorbidity index (0, 1, ≥2), insurance status (private, Medicare, Medicaid, uninsured, other, or missing), facility type (academic, nonacademic, or unknown), and region (Northeast, South, Midwest, West, or unknown). The Charlson Comorbidity index is designed to estimate long-term mortality, with a higher score indicating increasing risk of mortality. Clinical tumor, lymph node, and metastasis stage (I, II, III, IV, or missing) was categorized as defined by the *American Joint Committee on Cancer Staging Manual, 7th Edition* (used 2011 to 2017) or *American Joint Committee on Cancer Staging Manual, 8th Edition* (used 2018 to 2021). Additional details about covariates are available in the NCDB Participant User File 2022 Data Dictionary.^23^

Overall survival was determined in months from the time of diagnosis. Patients that were alive at the last point of follow-up or 5 years were censored. The cumulative percentage of missing data eligible for imputation was less than 2% across all variables and appeared to be missing at random. Therefore, a complete case analysis was performed.

### Statistical Analysis

Unadjusted 5-year survival was assessed by Kaplan-Meier analysis, with differences assessed by the log-rank test. For each of the 3 treatment groups, the median time to initiation of any therapy was similar: between 29 and 31 days after diagnosis. However, the CAM component of treatment tended to start later (34 days after diagnosis in the CAM group and 93 days in the combination group). Recognizing that by starting the CAM component of treatment later, the treatment cohorts receiving CAM (both CAM only and combination) may have a survival advantage in retrospective analysis (potential for immortal time bias), landmarking was performed at the longest median time to initiation of alternative therapy (3 months). These data are shown in eFigure 2 in Supplement 1. Stratification by key covariates, such as clinical summary stage, is not presented due to lack of power for significant head-to-head comparisons due to the small sample sizes of the alternative and combination groups.

Adjusted Cox proportional hazards model analysis was performed, controlling for age, race and ethnicity, Charlson Comorbidity index, insurance type, facility type, region, year of diagnosis, clinical summary stage, and median income. The traditional therapy cohort was considered the reference group, and adjusted hazard ratios (AHRs) were calculated. The proportion of patients receiving each of the different traditional treatment modalities (surgery, radiation, chemotherapy, immunotherapy, and endocrine therapy) was compared between the combination and traditional cohorts and compared with χ^2^ tests. Two-sided *P* < .05 was considered statistically significant. Data analysis was conducted from May 2025 to December 2025 SAS version 9.4 (SAS Institute Inc).

## Results

### Study Population

Overall, 2 169 202 female patients with breast cancer were identified. After excluding 11 983 patients with missing data, the sample included 2 157 219 (median [IQR] age, 62 [52-71] years), of whom 2 106 665 (97.6%) received traditional therapy, 273 (<0.1%) received CAM only, 568 (<0.1%) received a combination of traditional therapy and CAM, and 49 713 (2.3%) received no treatment (eFigure 1 in Supplement 1). Self-reported race and ethnicity included 6963 (0.3%) American Indian or Alaska Native women, 82 655 (3.8%) Asian women, 251 273 (11.7%) Black women, 136 431 (6.3%) Hispanic women, 5749 (0.3%) Native Hawaiian or Other Pacific Islander women, 1 644 328 (76.2%) White women, and 30 845 (1.4%) women of other race and ethnicity. Patients who did not receive treatment tended to be the older, with a median (IQR) age of 67 (55-78) years, followed by the traditional therapy group (median [IQR] age, 62 [52-71] years), while the combination group tended to be the youngest (median [IQR] age, 54 [44-63] years) (Table).

**Table.** Study Population Characteristics Stratified by Therapy Type

Characteristic	Patients, No. (%)^a^				*P* value
	CAM (n = 273)	Traditional therapy (n = 2 106 665)	Combination of traditional and CAM (n = 568)	No treatment (n = 49 713)	
Age, median (IQR)	57 (48-65)	62 (52-71)	54 (44-63)	67 (55-78)	NA
Race and ethnicity					
American Indian or Alaska Native	0	6793 (0.3)	0	170 (0.3)	<.001
Asian^b^	19 (7.0)	79 574 (3.8)	30 (5.3)	2032 (4.1)	
Black	30 (11.0)	242 984 (11.5)	40 (7.0)	8219 (16.5)	
Hispanic^c^	<10 (<3.6)^d^	132 761 (6.3)	34 (6.0)	3626 (7.3)	
Native Hawaiian or Other Pacific Islander	<10 (<3.6)^d^	5584 (0.3)	<10 (<1.8)^d^	145 (0.3)	
White	210 (76.9)	1 609 794 (76.4)	453 (79.8)	33 871 (68.1)	
Other^e^	<10 (<3.6)^d^	29 175 (1.4)	<10 (<1.8)^d^	1650 (3.3)	
Charlson Comorbidity index					
0	≥252 (≥92.3)^d^	1 730 960 (82.2)	≥568 (≥92.6)^d^	42 014 (84.5)	<.001
1	11 (4.0)	271 993 (12.9)	32 (5.6)	4502 (9.1)	
≥2	<10 (<3.6)^d^	103 712 (4.9)	<10 (<1.8)^d^	3197 (6.4)	
Insurance status					
Private	153 (56.0)	1 015 237 (48.2)	363 (63.9)	17 116 (34.4)	<.001
Uninsured	13 (4.8)	36 220 (1.7)	17 (2.9)	2223 (4.5)	
Medicaid	32 (11.7)	142 540 (6.8)	65 (11.4)	3839 (7.7)	
Medicare	67 (24.5)	861 289 (40.9)	114 (20.1)	24 396 (49.1)	
Other^f^	<10 (<3.6)^d^	23 195 (1.1)	<10 (<1.8)^d^	544 (1.1)	
Missing	<10 (<3.6)^d^	28 184 (1.3)	<10 (<1.8)^d^	1595 (3.2)	
Facility type					
Nonacademic medical center	198 (72.5)	1 395 754 (66.3)	328 (57.8)	34 616 (69.6)	<.001
Academic medical center	57 (20.9)	611 036 (29.0)	162 (28.5)	13 248 (26.7)	
Unknown	18 (6.6)	99 875 (4.7)	78 (13.7)	1849 (3.7)	
Region					
Northeast	26 (9.5)	429 128 (20.4)	59 (10.4)	10 820 (21.8)	<.001
South	60 (22.0)	738 303 (35.1)	122 (21.5)	19 853 (39.9)	
Midwest	66 (24.2)	484 709 (23.0)	116 (20.4)	8900 (17.9)	
West	103 (37.7)	354 650 (16.8)	193 (34.0)	8291 (16.7)	
Unknown	18 (6.6)	99 875 (4.)	78 (13.7)	1849 (3.7)	
Stage					
I	32 (11.7)	517 187 (24.6)	76 (13.4)	6672 (13.4)	<.001
II	90 (33.0)	454 303 (21.6)	185 (32.6)	8003 (16.1)	
III	30 (11.0)	115 777 (5.5)	63 (11.1)	2783 (5.6)	
IV	27 (9.9)	98 937 (4.7)	43 (7.6)	11 125 (22.4)	
Missing	94 (34.4)	920 461 (43.7)	201 (35.4)	21 130 (42.5)	

Abbreviation: CAM, complementary and alternative medicine.

^a^ Percentages may not total 100 due to rounding or censoring.

^b^ Asian includes Asian Indian or Pakistani not otherwise specified (NOS), Asian Indian, Pakistani, Micronesian NOS, Chamorro/Chamoru, Chinese, Fiji Islander, Filipino, Guamanian NOS, Hmong, Japanese, Kampuchean (Cambodian), Korean, Laotian, Melanesian NOS, New Guinean, Polynesian NOS, Samoan, Tahitian, Thai, Tongan, Vietnamese, and Other Asian including Asian NOS and Oriental NOS.

^c^ Hispanic included Cuban, Dominican, Mexican (including Chicano), Puerto Rican, South or Central American (except Brazilian), other specified Spanish/Hispanic origin includes European and excludes Dominican, Hispanic NOS, Latino NOS, Spanish NOS, and Spanish surname only.

^d^ Cells with fewer than 10 patients were censored in accordance with the National Cancer Database reporting guidelines.

^e^ Other includes racial or ethnic groups not included in other categories.

^f^ Other insurance includes TRICARE, Military, Veterans Affairs, and the Indian or Public Health Service.

### Sociodemographic Characteristics

Among the treatment groups, there were differences in sociodemographic attributes. For example, White women were the least likely to not receive treatment (33 871 of 644 328 [2.1%]) and the most likely to receive traditional therapies only (1 609 794 of 1 644 328 [97.9%]) (*P* < .001). Patients receiving CAM only were more likely to be healthy, with 252 or more of 273 patients (≥92.3%) having a Charlson Comorbidity index of 0 compared with 1 730 960 of 2 106 665 (82.2%) of patients receiving traditional therapies alone (*P* < .001) (Table). Private insurance was more common in the combination cohort (363 of 568 [63.9%]) compared with the traditional (1 015 237 of 2 106 665 [48.2%]) or no treatment (17 116 of 49 713 [34.4%]) cohorts (*P* < .001).

### Unadjusted Survival

**Figure 1.**
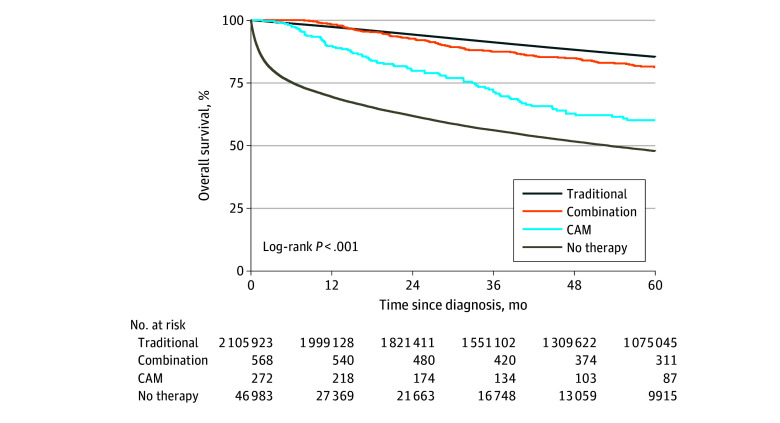
Survival Plot of Unadjusted Overall Survival Comparing Traditional Therapy, CAM Only, Combination Traditional Therapy and CAM, and No Therapy CAM indicates complementary and alternative medicine.

Traditional therapy was associated with the highest 5-year overall survival rates (85.4%; 95% CI, 85.3%-85.4%), followed by the combination group (81.2%; 95% CI, 77.5%-84.4%), the CAM-only group (60.1%; 95% CI, 53.9%-66.6%) and the no treatment group (47.8%; 95% CI, 47.3%-48.4%; log-rank *P* < .001) (Figure 1). Trends were generally similar after landmarking at the median time from diagnosis to initiation of CAM (eFigure 2 in the Supplement).

### Adjusted Survival

**Figure 2.**
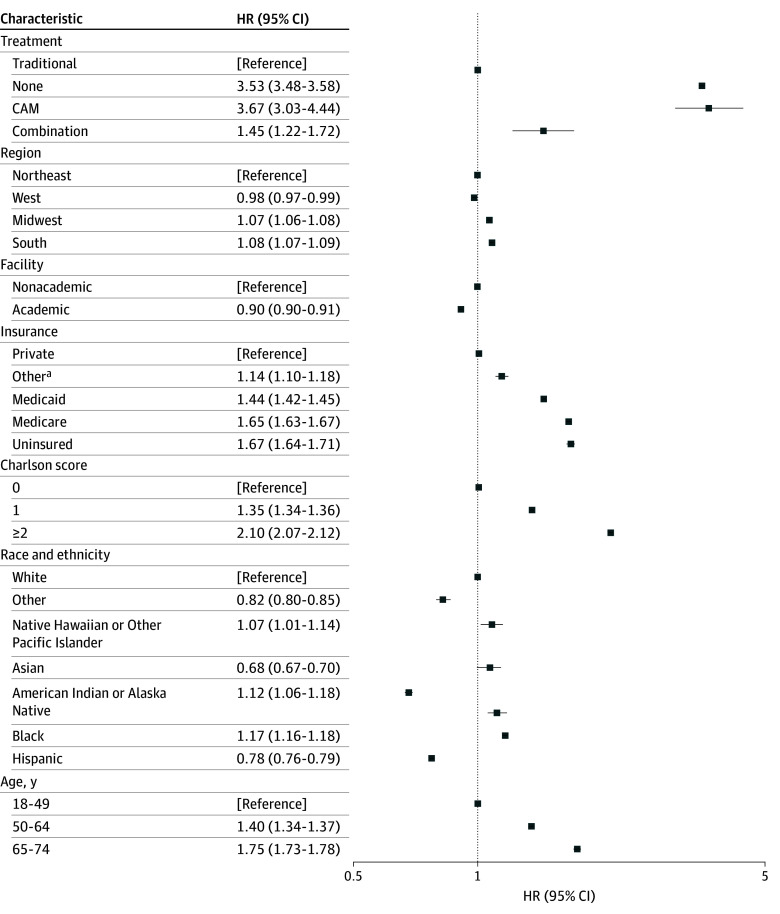
Forest Plot of Hazard Ratios for Female Patients With Breast Cancer Diagnosed From 2011 to 2021 HR indicates hazard ratio.

Adjusted Cox analysis was performed with patients receiving exclusively traditional therapy as the reference. Every treatment approach other than traditional therapy was associated with increased mortality (Figure 2). Patients who received CAM only had the highest mortality (AHR, 3.67; 95% CI, 3.03-4.44; *P* < .001) followed by untreated patients (AHR, 3.53; 95% CI, 3.48-3.58; *P* < .001). The combination of traditional and CAM was also associated with higher mortality (AHR, 1.45; 95% CI, 1.22-1.72; *P* < .001).

### Traditional Treatment Types Combined With CAM

**Figure 3.**
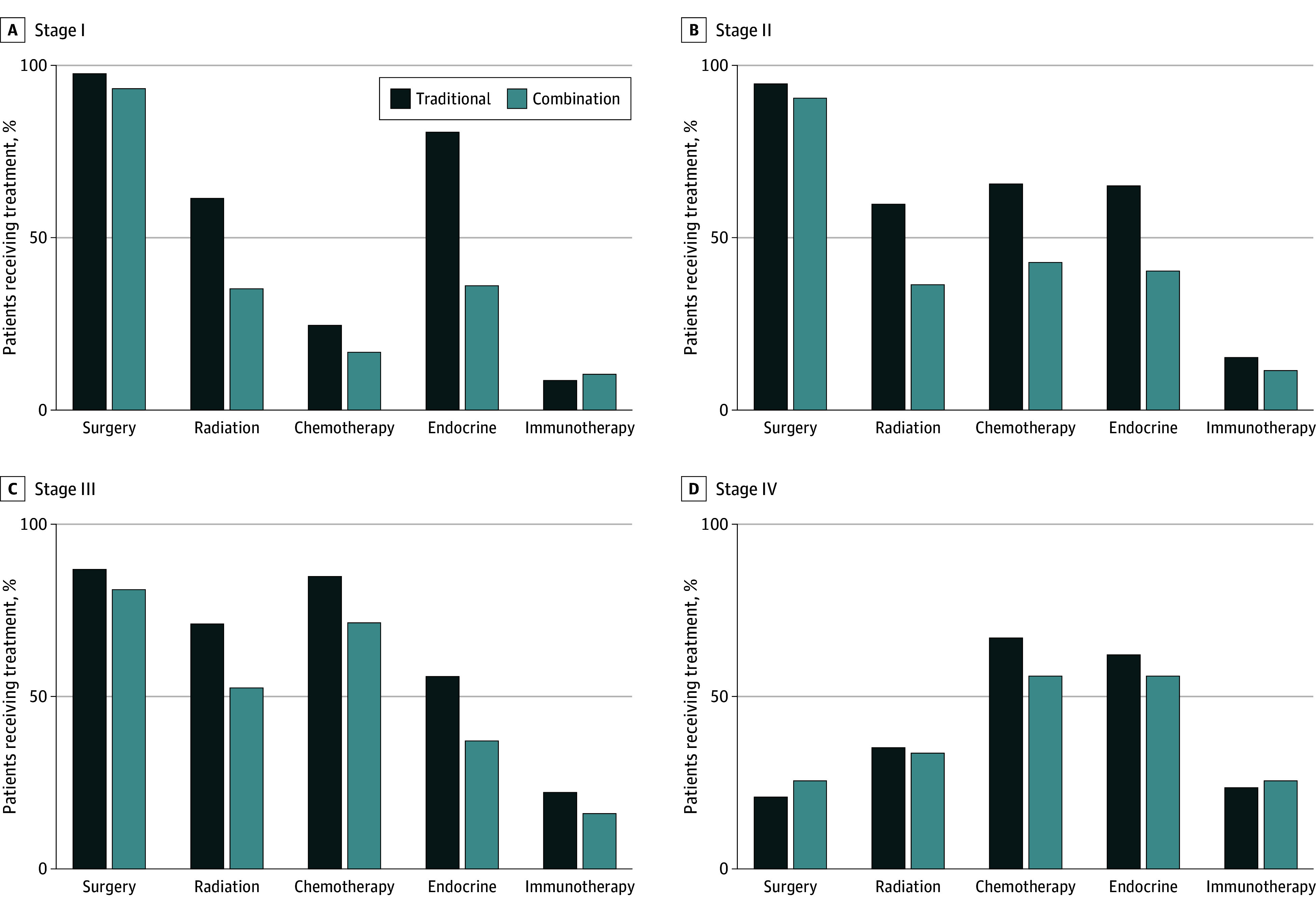
Bar Graph of Treatment Differences Among the Traditional Therapy and Combination Therapy Groups, Stratified by Disease Stage and Therapy Type

Recognizing the higher mortality associated with the combination of traditional therapy and CAM compared with traditional therapy only, the possibility that patients receiving CAM were skipping treatment was explored. The proportion of patients receiving each of the different traditional treatment modalities was compared among the combination and traditional therapy cohorts to assess elements of traditional therapy that might be omitted by patients in the combination cohort. Surgery was somewhat more common in the traditional therapy cohort than in the combination cohort across disease stages: stage I (504 861 of 516 536 [97.8%] vs 71 of 76 [93.4%], respectively; *P* = .01), stage II (429 578 of 453 604 [94.7%] vs 167 of 184 [90.8%], respectively; *P* < .001), and stage III (100 648 of 115 498 [87.1%] vs 51 of 63 [81.0%], respectively; *P* = .14) (Figure 3).

Differences in treatment patterns in the traditional therapy and combination groups were more pronounced for nonsurgical modalities. For example, radiation was used more often in the traditional therapy cohort compared with the combination cohort for stage I (312 534 of 507 783 [61.6%] vs 26 of 74 [35.1%], respectively; *P* < .001), stage II (256 850 of 431 696 [59.5%] vs 64 of 175 [36.6%], respectively; *P* < .001), and stage III (77 535 of 109 620 [70.7%] vs 32 of 61 [52.5%], respectively; *P* = .002). Receipt of chemotherapy and endocrine therapy was similar between the treatment groups across stages I to III. There were no statistically significant differences in receipt of any particular treatment modality for patients with stage IV breast cancer.

To better understand the consequences of omitting 1 form of traditional treatment, a sensitivity analysis was conducted as an unadjusted survival analysis for patients with stage II breast cancer, stratified by the receipt of 4 of traditional therapies (chemotherapy, radiation, surgery, and endocrine therapy) (eFigure 3 in Supplement 1). Patients with stage II breast cancer treated with a combination of traditional therapy and CAM had lower survival if they omitted radiation, endocrine therapy, or surgery, but the implications of omitting chemotherapy for survival in this cohort were not statistically significant.

## Discussion

In this cohort study, we found that the overall survival for patients with breast cancer who received CAM, either alone or in combination with traditional therapy, was shorter than that of patients who received traditional therapy. These findings are consistent with the results of a previous study that found that, when stratified by cancer types, receipt of CAM was associated with worse 5-year survival for breast, lung, and colorectal cancer.^24^ However, this earlier work had only 123 patients who received CAM for breast cancer and was conducted during an earlier time frame (2004 to 2013). Our study builds on previous findings, has a considerably larger sample size of patients who received CAM (841 patients), and further examines the survival of patients who received a combination of traditional therapies with CAM in subgroup analyses.

Adjusted survival analyses accounting for important differences in patient and tumor characteristics revealed that outcomes of combining traditional therapy and CAM were superior compared with no treatment, while outcomes of receiving CAM only were similar to those of no treatment. These findings highlight the importance of receiving at least some form of traditional treatment as being better than no treatment. These findings were observed despite adjustment for several factors known to be associated with differential survival, such as race and ethnicity,^25,26^ region,^27,28^ facility,^29,30^ and insurance status.^31,32^ The association of these socioeconomic determinants of health with survival were reflected the present study as well, where a lower comorbidity burden as well as treatment at an academic facility or with private insurance was associated with higher likelihood of survival.

The cohort of patients who received a combination of traditional therapy and CAM may have been avoiding certain forms of traditional treatment. While the use of surgery was largely preserved in the earlier stages of disease, the combination cohort appeared to be avoiding endocrine therapy, radiation, and chemotherapy. Omitting endocrine therapy may be associated with higher rates of recurrence,^33^ but can often be attributed to patients’ experiences with adverse effects^34^ or concerns about possible toxic effects before therapy initiation.^35^ Numerous risk factors have been identified as possible reasons for discontinuing or declining chemotherapy among patients with breast cancer, such as increased age and higher comorbidity burden.^36,37^ Receipt of radiation faces similar barriers, with age and low socioeconomic status associated with risk of declining or discontinuing treatment^38^; reasons cited for declining or stopping treatment include experienced or anticipated toxic effects and health care access concerns.^39,40^ The typically recommended delay between radiation and reconstructive surgery (due to wound healing concerns) may also play a role in patient reluctance to receive radiation.^41^

While the adjusted analysis identified a survival disadvantage among patients in the combination cohort, the precise consequence for any 1 patient is unclear. It is possible that some patients were able to forego 1 or more specific traditional modalities without a survival compromise. However, this possibility is confounded by the fact that some of the treatments missed are not clearly associated with survival advantages. Nonoperative approaches, such as radiation and endocrine therapy, can offer additional and more substantial benefits for risk reduction of recurrence,^42,43^ which could also be affected by substituting certain components of traditional therapy with CAM modalities. Given the available long-term end points for this study, we were not able to assess the possible implications for recurrence of substituting CAM for traditional therapies. However, to our knowledge, this is the first study to examine the combination of traditional therapy and CAM. Further study is warranted to clarify the implications of foregoing traditional treatment for breast cancer outcomes, as this information could enhance shared decision-making.

At the same time, because skipping traditional therapies appeared to be common in the combination cohort, a scenario we could not isolate was one in which the patients who fully complied with traditional treatments and then added CAM to the traditional treatment protocol. While the merits of traditional cancer treatments are clear, there are also indicators that treatments that would fall under the category of alternative therapies in the NCDB could have benefits during the cancer journey. Multiple studies have reported on the benefits of CAM approaches for symptom management and quality of life improvement in patients with cancer, including acupuncture,^44,45^ massage therapy,^46,47^ and mindfulness-based practices.^48,49^ Indeed, such therapies are increasingly studied and becoming incorporated into practice guidelines.^50,51^ Therefore, while traditional therapy should not be replaced by CAM, this study does not present any evidence to support or refute the role of CAM strictly as an adjunct to traditional therapy.

The relatively low rate of CAM use documented in the NCDB raises a concern that patients may not be discussing their interest in alternative treatments with their oncology teams. Specifically, several studies have estimated considerably higher rates of CAM—closer to 30%—in the breast cancer population.^9,44,52^ Because the NCDB is limited to capturing CAM use that is documented in the health record, patients may not be including their intentions to use treatment administered by nonmedical personal with their treatment teams. To date, one study found almost half of patients with breast cancer used CAM; however, many of them were not comfortable discussing this with their conventional treatment team because they did not believe that team had enough expertise on CAM.^52^ Another study found that while many patients choose to use CAM in addition to conventional treatment in the process of their cancer care, clinicians often lack the expertise to counsel patients on the evidence or lack thereof regarding CAM treatments.^44^ Inviting patients to share their interest in CAM may present an opportunity to enhance shared decision-making, particularly as patients may be planning to forego traditional treatments.

### Limitations

Beyond the traditional limitations that are associated with observational studies, this study has several limitations. The NCDB designation of alternative therapy likely misses many patients who choose CAM, some of which may have experienced meaningfully different outcomes. We suspect that many of these patients may fall within the group with no treatment, which has the worst survival. While we examined the use of traditional treatments across disease stages, we did not isolate standard treatment for each clinical scenario, and certain traditional treatments may only be indicated for a portion of the patients within a given stage category. We have attempted to balance this limitation by comparing the prevalence of traditional treatment use across the 3 cohorts, but we realize some of the differences may be attributed to differences in the clinical scenario within a stage group.

## Conclusions

In this cohort study of NCDB data on female patients with breast cancer use of CAM instead of traditional therapies was uncommon but was associated with a reduction in survival in breast cancer. Further study of CAM in this population is indicated.

## Notes

Supplement 1. **eFigure 1.** STROBE diagram for female breast cancer patients diagnosed between 2011 and 2021, excluding patients missing treatment information and patients missing information for traditional and CAM therapies **eFigure 2.** Unadjusted overall survival comparing traditional therapy, CAM, combination CAM & traditional therapy, no therapy, after having performed landmarking for each group at the median time interval from diagnosis to the initiation of alternative therapy **eFigure 3.** Treatment patterns in stage II breast cancer patients: traditional therapy was compared with combination CAM and traditional therapy, with or without each therapy types (radiation, chemotherapy, surgery, endocrine therapy)

Supplement 2.
Data Sharing Statement
