# Supplementary material for: Use of Complementary and Alternative Medicine in the Management of Breast Cancer
Source: JAMA Netw Open. 2026 Mar 2;9(3):e260337. doi: 10.1001/jamanetworkopen.2026.0337 (PMC12954545; doi:10.1001/jamanetworkopen.2026.0337)
Supplement: Supplement 1. — eFigure 1. STROBE diagram for female breast cancer patients diagnosed between 2011 and 2021, excluding patients missing treatment information and patients missing information for traditional and CAM therapies eFigure 2. Unadjusted overall survival comparing traditional therapy, CAM, combination CAM & traditional therapy, no therapy, after having performed landmarking for each group at the median time interval from diagnosis to the initiation of alternative therapy eFigure 3. Treatment patterns in stage II breast cancer patients: traditional therapy was compared with combination CAM and traditional therapy, with or without each therapy types (radiation, chemotherapy, surgery, endocrine therapy) [file jamanetwopen-e260337-s001.pdf]

Supplemental Online Content

Ayoade OF, Canavan ME, Caturegli G, Resio B, Berger E, Boffa DJ. Use of complementary and alternative medicine in the management of breast cancer. *JAMA Netw Open.* 2026;9(3): e260337. doi:10.1001/jamanetworkopen.2026.0337

eFigure 1. STROBE diagram for female breast cancer patients diagnosed between 2011 and 2021, excluding patients missing treatment information and patients missing information for traditional and CAM therapies

eFigure 2. Unadjusted overall survival comparing traditional therapy, CAM, combination CAM & traditional therapy, no therapy, after having performed landmarking for each group at the median time interval from diagnosis to the initiation of alternative therapy

eFigure 3. Treatment patterns in stage II breast cancer patients: traditional therapy was compared to combination with or without each of the following therapy types: radiation, chemotherapy, surgery, endocrine therapy

This supplemental material has been provided by the authors to give readers additional information about their work.

© 2026 Ayoade OF et al. *JAMA Network Open*.

eFigure 1. STROBE diagram for female breast cancer patients diagnosed between 2011 and 2021, excluding patients missing treatment information and patients missing information for traditional and CAM therapies

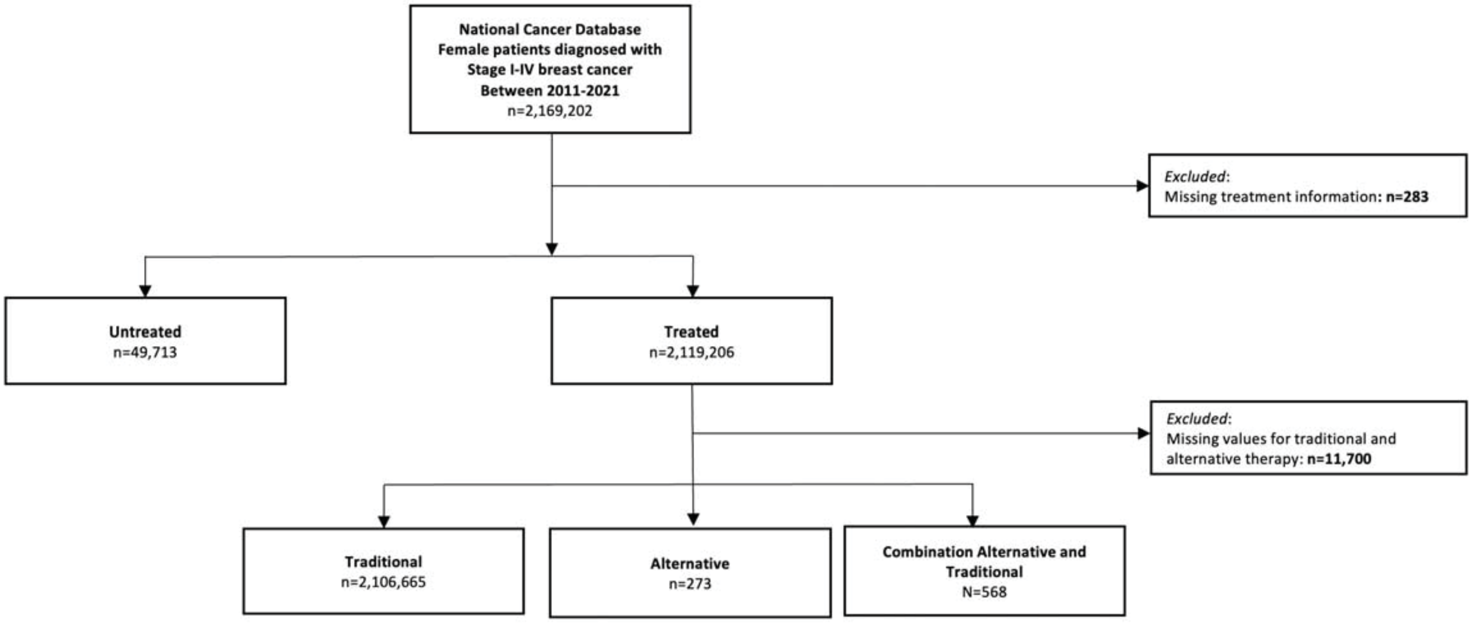

© 2026 Ayoade OF et al. *JAMA Network Open*.

eFigure 2. Unadjusted overall survival comparing traditional therapy, CAM, combination CAM & traditional therapy, no therapy, after having performed landmarking for each group at the median time interval from diagnosis to the initiation of alternative therapy

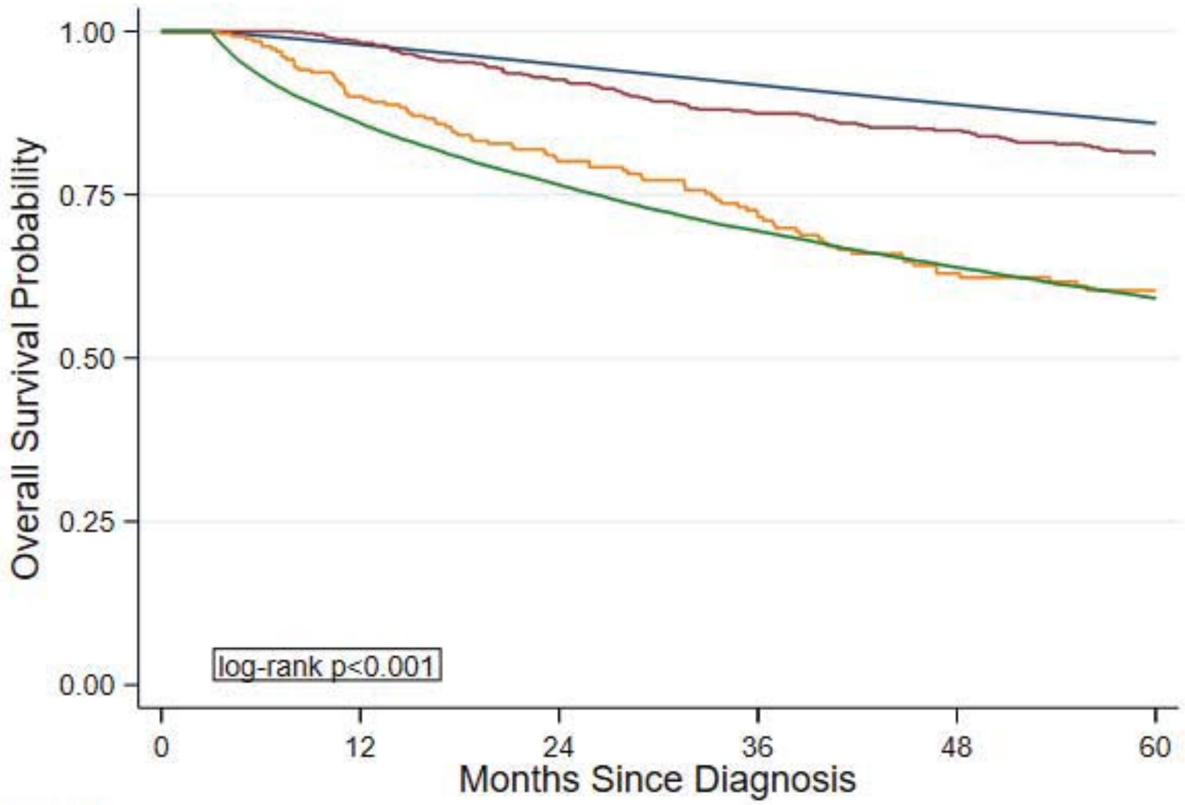

Number at risk

	0	12	24	36	48	60
Traditional	2082865	1999128	1821411	1551102	1309622	1075045
Combination	567	540	480	420	374	311
CAM	265	218	174	134	103	87
None	34364	27369	21663	16748	13059	9915

© 2026 Ayoade OF et al. *JAMA Network Open*.

eFigure 3. Treatment patterns in stage II breast cancer patients: traditional therapy was compared to combination with or without each of the following therapy types: radiation, chemotherapy, surgery, endocrine therapy

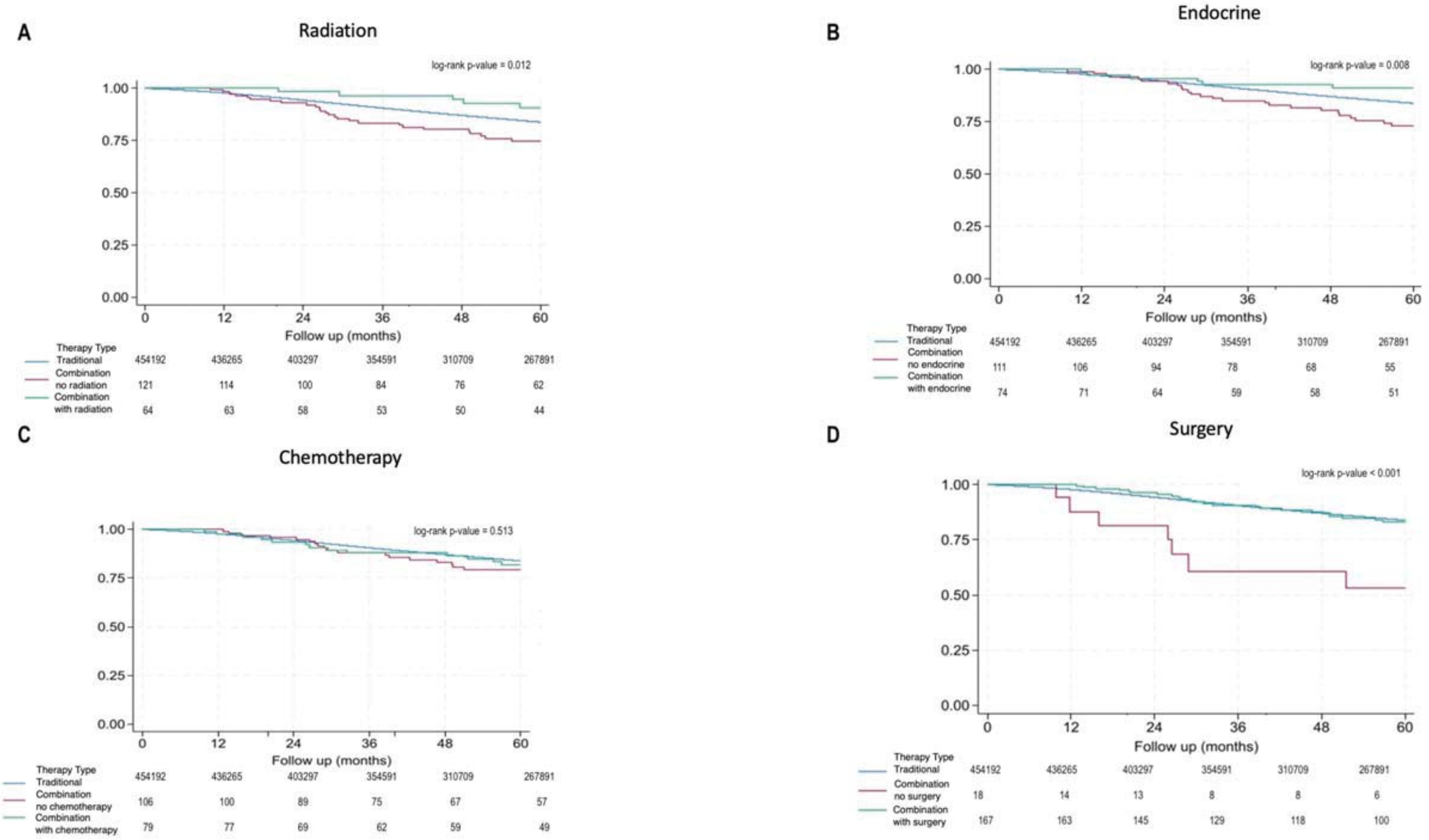

© 2026 Ayoade OF et al. *JAMA Network Open*.
