# Supplementary material for: Use of Complementary and Alternative Medicine in the Management of Breast Cancer
Source: JAMA Netw Open. 2026 Mar 2;9(3):e260337. doi: 10.1001/jamanetworkopen.2026.0337 (PMC12954545; doi:10.1001/jamanetworkopen.2026.0337)
Supplement: Supplement 2. — Data Sharing Statement [file jamanetwopen-e260337-s002.pdf]

Data Sharing Statement

Ayoade. Use of Complementary and Alternative Medicine in the Management of Breast Cancer. *JAMA Netw Open*. Published March 02, 2026.
doi:10.1001/jamanetworkopen.2026.0337

Data
Data available: No

Additional Information
Explanation for why data not available: The National Cancer Database permits sharing of data only after the approval of a central request with an associated data use agreement.
